# Molecular and morphological evidence for three species of *Diplostomum* (Digenea: Diplostomidae), parasites of fishes and fish-eating birds in Spain

**DOI:** 10.1186/s13071-014-0502-x

**Published:** 2014-11-12

**Authors:** Ana Pérez-del-Olmo, Simona Georgieva, Héctor J Pula, Aneta Kostadinova

**Affiliations:** Unidad de Zoología Marina, Institut Cavanilles de Biodiversitat i Biologia Evolutiva, Parc Científic, Universitat de València, PO Box 22085, Valencia, 46071 Spain; Institute of Parasitology, Biology Centre of the Academy of Sciences of the Czech Republic, Branišovská 31, 370 05 České Budějovice, Czech Republic; Faculty of Science, University of South Bohemia, Branišovská 31, 370 05 České Budějovice, Czech Republic; Piscifactoría Vegas del Guadiana, Gobierno de Extremadura, Antigua Ctra. N-V, km. 391, 7 s/n, C.P. 06195, Villafranco del Guadiana, Badajoz Spain

**Keywords:** *Diplostomum spathaceum*, *Diplostomum pseudospathaceum*, Lens metacercariae, Freshwater fish, Gulls, Spain, *Cox*1, ITS1-5.8S-ITS2

## Abstract

**Background:**

Recent molecular studies have revealed high species diversity of *Diplostomum* in central and northern Europe. However, our knowledge of the distribution of *Diplostomum* spp. in the southern distributional range in Europe of the snail intermediate hosts (*Lymnaea stagnalis* and *Radix* spp.) is rather limited. This study aims to fill this gap in our knowledge using molecular and morphological evidence.

**Methods:**

Nineteen fish species and six fish-eating bird species were sampled opportunistically in three regions (Catalonia, Extremadura and Aragon) in Spain. All isolates of *Diplostomum* spp. were characterised morphologically and molecularly. Partial sequences of the barcode region of the *cox*1 mitochondrial gene and complete sequences of the ribosomal ITS1-5.8S-ITS2 gene cluster were used for molecular identification of the isolates.

**Results:**

Integrated morphological and molecular analyses demonstrated the presence of three species among the larval and adult isolates of *Diplostomum* spp. sampled in Spain: *Diplostomum spathaceum* (in fish and birds), *D. pseudospathaceum* (in birds) and *Diplostomum* sp. (in fish) referred to as Clade Q *sensu* Georgieva *et al*. (Int J Parasitol, 43:57–72, 2013). We detected ten *cox*1 haplotypes among the isolates of *D. spathaceum* with only one haplotype shared with adult isolates from central and northern Europe. No specific geographic pattern of the distribution of the novel haplotypes was found.

**Conclusion:**

This first molecular exploration of the diversity of *Diplostomum* spp. in southern Europe indicates much lower species richness compared with the northern regions of Europe.

## Background

*Diplostomum* von Nordmann, 1832 is a relatively large genus of widely distributed digeneans with three-host life-cycles involving lymnaeid snails and fish as intermediate hosts and fish-eating birds (predominantly gulls) as definitive hosts. There are 41 nominal species described within the Palaearctic, mainly from Europe (see [1] for details). However, treatment of the data on the geographic and host ranges of *Diplostomum* spp. have long been hindered by taxonomic and identification problems concerning all life-cycle stages.

The use of molecular markers has proved to be valuable and more efficient than experimental approaches in elucidating parasite life-cycles by linking larvae with adults, e.g. [1-5]. The mitochondrial cytochrome *c* oxidase subunit 1 (*cox*1) barcode region was found to be suitable for this goal as well as for the identification and recognition of cryptic species diversity within *Diplostomum* [1,6,7].

Recent molecular studies linking *cox*1 and ITS1-5.8S-ITS2 sequences for larval and adult isolates, which were identified based on parasite morphology, have revealed high species diversity of *Diplostomum* in central and northern Europe [1,7]. However, our knowledge of the distribution of *Diplostomum* spp. in the southern distributional range in Europe of the snail intermediate hosts (*Lymnaea stagnalis* and *Radix* spp.) is rather limited. Virtually no data exist for infections with *Diplostomum* spp. in the intermediate and definitive hosts in southern Europe. In Spain, two species have been recorded in populations of the gull definitive hosts. *Diplostomum spathaceum* was reported in four out of 324 yellow-legged gulls referred to as “*Larus cachinnans*” [8] and “*Larus michahellis*” [9] in Galicia and *D. pseudospathaceum* was recorded in one of 122 “*L. cachinnans*” from Medes Islands [10,11]. Similarly, there is a lack of data from the intermediate fish hosts; only unidentified metacercariae of *Diplostomum* sp. were reported in *Anguilla anguilla* in the Rivers Ulla and Tea in Galicia [12].

In this study, we used the molecular framework and the recently generated genetic datasets for Nearctic and Palaearctic species of the genus [1,6,13] to investigate species diversity of *Diplostomum* in birds and fishes sampled opportunistically in three regions in the northern and southern Spain. We provide the first molecular evidence associated with descriptions of the hologenophores *sensu* Pleijel *et al*. [14] for three species of *Diplostomum*.

## Methods

### Sample collection and processing

**Figure 1:**
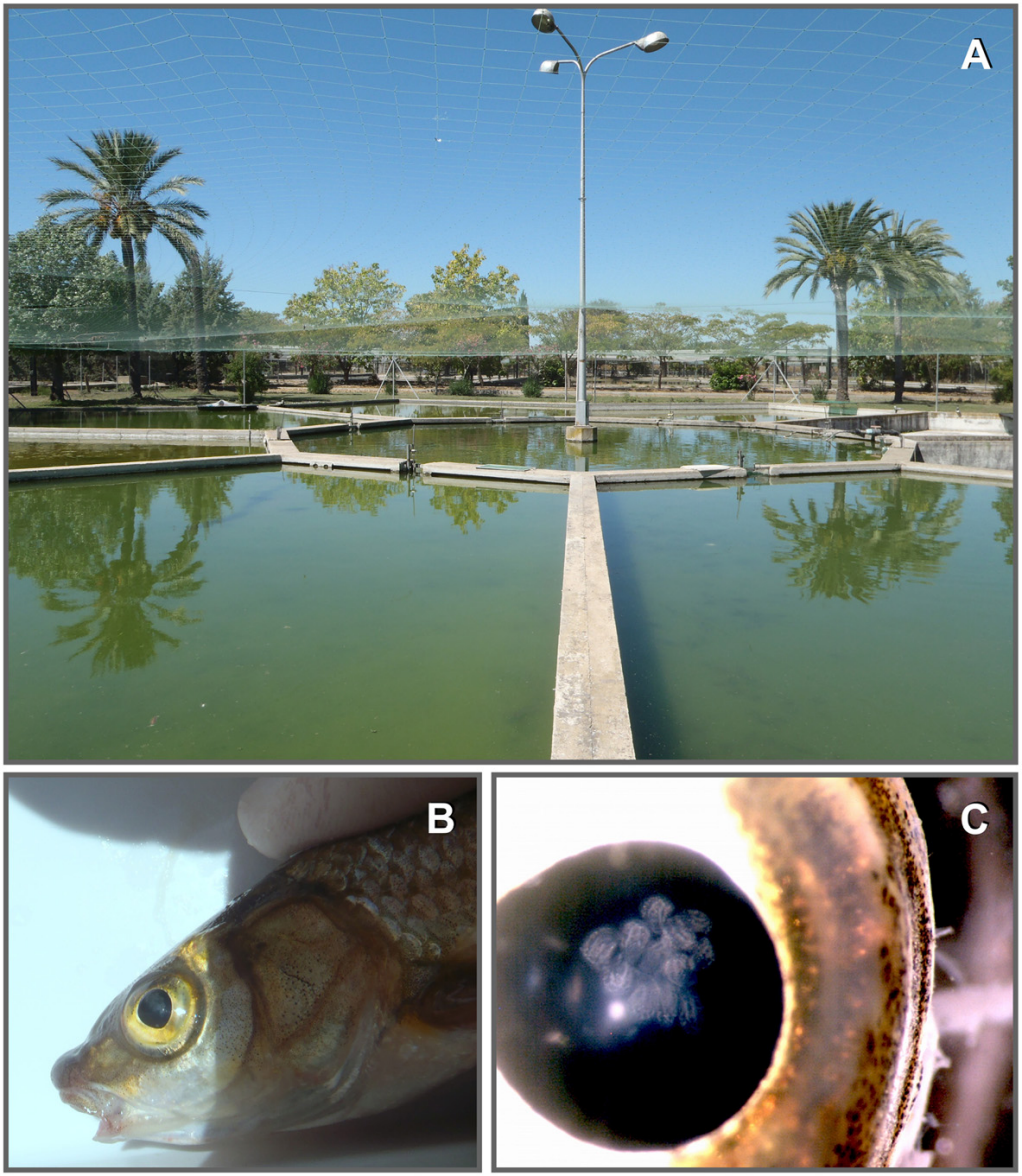
**Focus of infection with** ***D. spathaceum*** **at the Aquaculture Centre in Villafranco del Guadiana. A**, Pool system for culturing *Pseudochondrostoma willkommi* at the Aquaculture Centre in Villafranco del Guadiana; **B**, *P. willkommii* infected with large numbers of lens-dwelling metacercariae of *Diplostomum spathaceum*; **C**, Eye of *P. willkommii* with lens capsule close to rupture due to the large numbers of metacercariae of *D. spathaceum*.

An opportunistic sampling strategy was adopted for this study, which was focused on examination of a diverse array of hosts rather than large samples of a single host species. Table 1 provides a list of the fish hosts and localities in different regions in Spain. Fish were obtained in collaboration with the regional governments of Extremadura, Aragón and Catalunya. A total of 230 fish belonging to 19 species and 10 families was examined in 2012 for the presence of eye dwelling metacercariae. The samples of *Pseudochondrostoma willkommi* and *Salmo trutta* collected in Villafranco del Guadiana and Jerte were obtained from aquiculture centres of the regional government of Extremadura whereas the remaining fish species/samples were collected in rivers. The largest number of individuals and species was collected in the Ebro Delta. The aquaculture system in Villafranco del Guadiana Aquaculture Centre comprises a central octagonal pool (depth 1 m; surface c.100 m^2^) surrounded by a group of pentagonal pools (depth <1 m; surface c.100 m^2^) (Figure 1A). The central pool is used for culturing mature breeders of *P. willkommi* of different ages whereas the peripheral pools are used for fish fry for up to two seasons; the latter are transferred to the central pool after reaching maturity. All pools are covered with nets to decrease predation by fish-eating birds and have an open water circulation system with a steady flow of 20 L/min. Although efforts are made to keep the water quality within the accepted ranges, the degree of eutrophication is high. Pools have been completely dried on various occasions but soon afterwards were repopulated by freshwater snails.

**Table 1:** **Summary data for the fish species examined/infected with** ***Diplostomum*** **spp.**

**Fish species**	**Fish family**	**Locality**	**Date of collection**	**No. examined (infected)**	**Total length (range, mm)**
*Carassius auratus (L.)	Cyprinidae	Ebro Delta^a^	18.ii.2012	2	121 − 248
*Cyprinus carpio L.	Cyprinidae			13 (1)	290 − 379
*Silurus glanis L.	Siluridae			2	440 − 460
*Pseudorasbora parva (Temminck & Schlegel)	Cyprinidae			15	45 − 103
*Lepomis gibbosus (L.)	Centrarchidae			1	52
*Liza ramada* (Risso) juv.	Mugilidae			10	90 − 183
*Misgurnus anguillicaudatus (Cantor)	Cobitidae			15 (1)	50 − 128
*Anguilla anguilla* (L.)	Anguillidae	Ebro Delta^a^	17.v.2012	5	158 − 255
*Atherina boyeri* Risso	Atherinidae			10	34 − 44
*Cyprinus carpio L.	Cyprinidae			1	192
*Gambusia holbrooki Girard	Poeciliidae			18	24 − 50
*Liza ramada* (Risso) juv.	Mugilidae			1	58
*Lepomis gibbosus (L.)	Centrarchidae			14	43 − 65
*Misgurnus anguillicaudatus (Cantor)	Cobitidae			16 (2)	52 − 122
*Pomatoschistus microps* (Krøyer)	Gobiidae			1	32
*Pseudorasbora parva (Temminck & Schlegel)	Cyprinidae			27	49 − 79
*Silurus glanis L.	Siluridae			1 (1)	409
*Tropidophoxinellus alburnoides* (Steindachner)	Cyprinidae	River Albarragena^b^	21.ii.2012	4	57 − 89
*Tropidophoxinellus alburnoides* (Steindachner)	Cyprinidae	River Luorianilla^b^	06.vi.2012	8	55 − 75
*Pseudochondrostoma willkommii* (Steindachner)	Cyprinidae	Villafranco del Guadiana^b^	06.iii.2012	10 (10)	235 − 262
*Salmo trutta* L.	Salmonidae	Jerte^c^	07.iii.2012	3	262 − 291
*Parachondrostoma miegii* (Steindachner)	Cyprinidae	River Piedra^d^	24.ix.2012	5	139 − 177
*Oncorhynchus mykiss* (Walbaum)	Salmonidae			2	170 − 195
*Squalius pyrenaicus* (Günther)	Cyprinidae			10	84 − 135
*Salmo trutta* L.	Salmonidae	Lake Espejo^d^	24.ix.2012	2	490 − 497
*Luciobarbus graellsii* (Steindachner)	Cyprinidae			3	236 − 405
*Oncorhynchus mykiss* (Walbaum)	Salmonidae			1	441
*Salmo trutta* L.	Salmonidae	River Aragon^d^	25.ix.2012	12	70 − 188
*Salmo trutta* L.	Salmonidae	River Ara^e^	25.ix.2012	12	68 − 146
*Gobio lozanoi* Doadrio & Madeira	Cyprinidae	River Cinca^e^	25.ix.2012	1	53
*Gambusia holbrooki Girard	Poeciliidae			5	21 − 29

*Invasive species are marked with a star; ^a^Tarragona; ^b^Badajoz; ^c^Caceres; ^d^Zaragoza; ^e^Huesca.

A total of 31 fish eating birds were obtained from bird recovery centres in Catalunya (Spain) in 2012 in order to obtain adult specimens of *Diplostomum* (Table 2). Six species of birds of four families were examined: (i) Laridae [*Larus ridibundus* L., *Larus argentatus michahellis* Naumann]; (ii) Sternidae [*Sterna sandvicensis* Latham]; (iii) Ardeidae [*Ardea cinerea* L. and *Ixobrychus minutus* (L.)]; (iv) Phalacrocoracidae [*Phalacrocorax aristotelis* (L.)]. The largest number of birds was obtained from the Ebro Delta.

**Table 2:** **Summary data for the bird species examined/ infected with** ***Diplostomum*** **spp.**

**Bird species**	**Collection site**	**No. examined (infected)**
*Larus argentatus michahellis* Naumann	Ebro Delta (Tarragona)	6 (2)
*Larus argentatus michahellis* Naumann	Barcelona	2
*Larus argentatus michahellis* Naumann	Alella (Barcelona)	1
*Larus argentatus michahellis* Naumann	Sabadell (Barcelona)	1
*Larus argentatus michahellis* Naumann	Empuria Brava (Girona)	1
*Larus argentatus michahellis* Naumann	Figueres (Girona)	1
*Larus argentatus michahellis* Naumann	Roses (Girona)	2
*Larus argentatus michahellis* Naumann	Tarragona	1
*Larus argentatus michahellis* Naumann	Cambrils (Tarragona)	1
*Larus ridibundus* L.	Ebro Delta (Tarragona)	5 (3)
*Larus ridibundus* L.	Cunit (Tarragona)	1 (1)
*Sterna sandvicensis* (Latham)	Roda de Bará (Tarragona)	1
*Phalacrocorax aristotelis* (L.)	Tarragona	1
*Ardea cinerea* L.	Ebro Delta (Tarragona)	5
*Ixobrychus minutus* (L.)	Ebro Delta (Tarragona)	2

All metacercariae were dissected out from fresh fish, fixed in hot saline solution and preserved in molecular biology grade ethanol whereas all adult worms were collected from birds found dead and frozen until necropsy; these were also preserved in molecular grade ethanol. The morphology of the larval and adult stages of *Diplostomum* spp. was studied on live and fixed material from series of photomicrographs made for each isolate with a digital camera of an Olympus BX51 microscope prior to sequencing; measurements were taken from the digital images with the aid of Quick Photo Camera 2.3 image analysis software. The structure of the secondary excretory system was reconstructed from serial microphotographs and the number of excretory concretions was counted.

All measurements in the descriptions and tables are in micrometres and are presented as the range followed by the mean in parentheses.

### Sequence generation

Total genomic DNA was isolated from single ethanol-fixed adult individuals using the Chelex method (see [15] for details). Partial fragments of the barcode region of the *cox*1 mitochondrial gene [16] were obtained by polymerase chain reaction (PCR) amplifications using Ready-To-Go PCR beads (GE Healthcare, UK) and the diplostomid-specific PCR primers Plat-diploCOX1F (5′-CGT TTR AAT TAT ACG GAT CC-3′) and Plat-diploCOX1R (5′-AGC ATA GTA ATM GCA GCA GC-3′) designed by Moszczynska *et al*. [16] (see [1] for details). PCR amplifications of the ITS1-5.8S-ITS2 gene cluster were performed as above using the primers D1 (forward: 5′-AGG AAT TCC TGG TAA GTG CAA G-3′) and D2 (reverse: 5′-CGT TAC TGA GGG AAT CCT GGT-3′) and thermocycling conditions of Galazzo *et al*. [17].

PCR amplicons were purified using a QIAquick PCR purification kit (Qiagen Ltd, UK) and sequenced directly from both strands using the PCR primers (*cox*1) and the primers from [18]: BD1 (forward: 5′-GTC GTA ACA AGG TTT CCG TA-3′) and BD2: (reverse: 5′-TAT GCT TAA ATT CAG CGG GT-3′) (ITS1-5.8S-ITS2) with ABI BigDye chemistry (ABI Perkin-Elmer, UK), alcohol-precipitated, and run on an ABI Prism 3130 x 1 automated sequencer. Contiguous sequences were assembled with MEGA v5 [19] and submitted to GenBank (details and accession numbers are shown in Table 3).

**Table 3:** **Summary data for the isolates of** ***Diplostomum*** **spp. from fishes and birds collected in Spain and used for generation of the** ***cox*** **1 and ITS1-5.8S-ITS2 sequences**

**Species**	**Life-cycle stage** ^**a**^	**Isolate**	**Haplotype**	**Host**	**Locality**	**GenBank accession numbers**	
						***cox*** **1**	**ITS1-5.8S-ITS2**
*Diplostomum* sp. (Clade Q)	M	CCED	−	*Cyprinus carpio*	Ebro Delta	KP025770	KP025788
*Diplostomum pseudospathaceum*	A	LRED1	−	*Larus ridibundus*	Ebro Delta	KP025771	JX986854^b^
*Diplostomum spathaceum*	A	LCED1	1	*Larus argentatus michahellis*	Ebro Delta	KP025772	–
*Diplostomum spathaceum*	A	LCED2	6	*Larus argentatus michahellis*	Ebro Delta	KP025773	–
*Diplostomum spathaceum*	A	LCED3	4	*Larus argentatus michahellis*	Ebro Delta	KP025774	–
*Diplostomum spathaceum*	A	LRC	3	*Larus ridibundus*	Cunit	KP025775	KP025789
*Diplostomum spathaceum*	A	LRED2	10	*Larus ridibundus*	Ebro Delta	KP025776	–
*Diplostomum spathaceum*	A	LRED3	8	*Larus ridibundus*	Ebro Delta	KP025777	–
*Diplostomum spathaceum*	M	MAED1	4	*Misgurnus anguillicaudatus*	Ebro Delta	KP025778	KP025790
*Diplostomum spathaceum*	M	MAED2	2	*Misgurnus anguillicaudatus*	Ebro Delta	KP025779	KP025791
*Diplostomum spathaceum*	M	PWVG1	5	*Pseudochondrostoma willkommii*	Villafranco del Guadiana	KP025780	–
*Diplostomum spathaceum*	M	PWVG2	4	*Pseudochondrostoma willkommii*	Villafranco del Guadiana	KP025781	KP025792
*Diplostomum spathaceum*	M	PWVG3	7	*Pseudochondrostoma willkommii*	Villafranco del Guadiana	KP025782	KP025793
*Diplostomum spathaceum*	M	PWVG4	1	*Pseudochondrostoma willkommii*	Villafranco del Guadiana	KP025783	–
*Diplostomum spathaceum*	M	PWVG5	9	*Pseudochondrostoma willkommii*	Villafranco del Guadiana	KP025784	–
*Diplostomum spathaceum*	M	PWVG6	3	*Pseudochondrostoma willkommii*	Villafranco del Guadiana	KP025785	–
*Diplostomum spathaceum*	M	PWVG7	7	*Pseudochondrostoma willkommii*	Villafranco del Guadiana	KP025786	–
*Diplostomum spathaceum*	M	SGED	6	*Silurus glanis*	Ebro Delta	KP025787	–

^a^M, metacercaria, A, adult; ^b^ITS sequence identical with JX986854 of Georgieva *et al*. [1].

### Alignments and data analysis

The newly-generated and published sequences were aligned together with MUSCLE implemented in MEGA v5; *cox*1 sequences were aligned with reference to the amino acid translation, using the echinoderm and flatworm mitochondrial code [20]. The *cox*1 alignment (410 nt; 46 sequences) comprised the 18 newly-generated (Table 3) and 28 published sequences, the latter including 1 − 5 representative sequences per species/lineage identified in previous studies in Europe [1,13]; see Table 4 for details. The ITS1-5.8S-ITS2 alignment (997 nt; 35 sequences) comprised seven new sequences for Spanish isolates sub-sampled within the *cox*1-derived clades and 29 published sequences, representative for the species/lineages sequenced in Europe [1,13] and Canada [6,17] (for details see Table 4). Sequences for *Tylodelphys clavata* were used as outgroups.

**Table 4:** **Summary data for the isolates of** ***Diplostomum*** **spp. retrieved from GenBank**

**Trematode species**	**Isolate**	**Life-cycle stage** ^**a**^	**Host species**	**Locality**	**Accession No. (** ***cox*** **1)**	**Accession No. (ITS1-5.8S-ITS2)**	**Reference**
‘*Diplostomum baeri*’ 1	STR3	M	*Salmo trutta fario*	Germany: River Ruhr (Henne)	JX986862	JX986837	Georgieva *et al*. [1]
‘*Diplostomum baeri*’ 1	STL1	M	*Salmo trutta fario*	Germany: River Lenne	JX986863	-	Georgieva *et al*. [1]
‘*Diplostomum baeri*’ 1	STR4	M	*Salmo trutta fario*	Germany: River Ruhr (Henne)	JX986864		Georgieva *et al*. [1]
‘*Diplostomum baeri*’ 1	STL2	M	*Salmo trutta fario*	Germany: River Lenne	JX986865	-	Georgieva *et al*. [1]
‘*Diplostomum baeri*’ 1	STR7	M	*Salmo trutta fario*	Germany: River Ruhr (Henne)	JX986869	-	Georgieva *et al*. [1]
*Diplostomum baeri*	PF5D3	M	*Perca fluviatilis*	Germany: Lake Constance	JQ639195	-	Behrmann-Godel [13]
*Diplostomum baeri*	PF15D9	M	*Perca fluviatilis*	Germany: Lake Constance	JQ639193	-	Behrmann-Godel [13]
*Diplostomum baeri*	PF15D4	M	*Perca fluviatilis*	Germany: Lake Constance	JQ639187	-	Behrmann-Godel [13]
*Diplostomum baeri*	PF8D7	M	*Perca fluviatilis*	Germany: Lake Constance	JQ639191	-	Behrmann-Godel [13]
*Diplostomum baeri*	PF6D3	M	*Perca fluviatilis*	Germany: Lake Constance	JQ639189	-	Behrmann-Godel [13]
*Diplostomum baeri*	–	A	*Larus delawarensis* (exp.)^b^	Canada	-	AY123042	Galazzo *et al*. [17]
*Diplostomum huronense*	–	A	*Larus delawarensis* (exp.)^b^	Canada	-	AY123044	Galazzo *et al*. [17]
*Diplostomum huronense*	D.LL.IVT.Cc.3 F.1	M	*Catostomus commersoni*	Canada	-	GQ292513	Locke *et al*. [6]
*Diplostomum indistinctum*	D.RL.D.Cc.1.2	M	*Catostomus commersoni*	Canada		GQ292508	Locke *et al*. [6]
‘*Diplostomum mergi*’ 1	RAH1	C	*Radix auricularia*	Germany: Hengsteysee	JX986873	JX986838	Georgieva *et al*. [1]
‘*Diplostomum mergi*’ 2	RAH2	C	*Radix auricularia*	Germany: Hengsteysee	JX986874	-	Georgieva *et al*. [1]
‘*Diplostomum mergi*’ 2	RAH3	C	*Radix auricularia*	Germany: Hengsteysee	JX986875	JX986839	Georgieva *et al*. [1]
‘*Diplostomum mergi*’ 2	RAH4	C	*Radix auricularia*	Germany: Hengsteysee	JX986876	-	Georgieva *et al*. [1]
‘*Diplostomum mergi*’ 3	GGR2	M	*Gobio gobio*	Germany: River Ruhr (Henne)	JX986877	JX986840	Georgieva *et al*. [1]
‘*Diplostomum mergi*’ 3	STR10	M	*Salmo trutta fario*	Germany: River Ruhr (Henne)	JX986878	JX986841	Georgieva *et al*. [1]
‘*Diplostomum mergi*’ 3	STR11	M	*Salmo trutta fario*	Germany: River Ruhr (Henne)	JX986879	-	Georgieva *et al*. [1]
‘*Diplostomum mergi*’ 3	STR12	M	*Salmo trutta fario*	Germany: River Ruhr (Henne)	JX986880	-	Georgieva *et al*. [1]
‘*Diplostomum mergi*’ 3	GGR3	M	*Gobio gobio*	Germany: River Ruhr (Henne)	-	JX986842	Georgieva *et al*. [1]
‘*Diplostomum mergi*’ 3	GGR4	M	*Gobio gobio*	Germany: River Ruhr (Henne)	-	JX986843	Georgieva *et al*. [1]
‘*Diplostomum mergi*’ 3	STR15	M	*Salmo trutta fario*	Germany: River Ruhr (Henne)	JX986886	-	Georgieva *et al*. [1]
*Diplostomum mergi*	RR45	M	*Rutilus rutilus*	Germany: Lake Constance	JQ639178	-	Behrmann-Godel [13]
*Diplostomum mergi*	RR43	M	*Rutilus rutilus*	Germany: Lake Constance	JQ639177	-	Behrmann-Godel [13]
*Diplostomum mergi*	RA97	C	*Radix auricularia*	Germany: Lake Constance	JQ639179	JQ665458	Behrmann-Godel [13]
*Diplostomum paracaudum*	CL100	M	*Coregonus lavaretus*	Germany: Lake Constance	-	JQ665457	Behrmann-Godel [13]
*Diplostomum pseudospathaceum*	LCT3	A	*Larus cachinnans*	Czech Republic: near Tovačov	JX986896	JX986849	Georgieva *et al*. [1]
*Diplostomum pseudospathaceum*	LSB2	C	*Lymnaea stagnalis*	Germany: Baldeneysee	-	JX986850	Georgieva *et al*. [1]
*Diplostomum pseudospathaceum*	LSH1	C	*Lymnaea stagnalis*	Germany: Harkortsee	-	JX986851	Georgieva *et al*. [1]
*Diplostomum pseudospathaceum*	GAH6	M	*Gasterosteus aculeatus*	Germany: Hengsteysee	-	JX986852	Georgieva *et al*. [1]
*Diplostomum pseudospathaceum*	LAG2	A	*Larus argentatus*	Poland: near Gdańsk	JX986904	JX986853	Georgieva *et al*. [1]
*Diplostomum pseudospathaceum*	LCT4	A	*Larus cachinnans*	Czech Republic: near Tovačov	JX986905	JX986854	Georgieva *et al*. [1]
*Diplostomum pseudospathaceum*	GC87	M	*Gymnocephalus cernuus*	Germany: Lake Constance	-	JQ665456	Behrmann-Godel [13]
*Diplostomum spathaceum*	LCT1	A	*Larus cachinnans*	Czech Republic: near Tovačov	JX986887	JX986844	Georgieva *et al*. [1]
*Diplostomum spathaceum*	RAH6	C	*Radix auricularia*	Germany: Hengsteysee		JX986846	Georgieva *et al*. [1]
*Diplostomum spathaceum*	RAH5	C	*Radix auricularia*	Germany: Hengsteysee		JX986845	Georgieva *et al*. [1]
*Diplostomum spathaceum*	LAG1	A	*Larus argentatus*	Poland: near Gdańsk	JX986892	JX986847	Georgieva *et al*. [1]
*Diplostomum spathaceum*	LCT2	A	*Larus cachinnans*	Czech Republic: near Tovačov	JX986895	JX986848	Georgieva *et al*. [1]
*Diplostomum* sp. 1 SAL-2008	D.IN.SSO.Ld.2 F.6	A	*Larus delawarensis* (exp.)^b^	Canada	-	GQ292519	Locke *et al*. [6]
*Diplostomum* sp. 2 SAL-2008	D.BR.S.B.20.1	M	*Pimephales notatus*	Canada	-	GQ292505	Locke *et al*. [6]
*Diplostomum* sp. 3 SAL-2008	D.RL.B08.Ms.1 F.1	M	*Micropterus salmoides*	Canada	-	GQ292511	Locke *et al*. [6]
*Diplostomum* sp. 4 SAL-2008	D.IN.SSO.Ld.2 F.10	A	*Larus delawarensis*	Canada	-	GQ292520	Locke *et al*. [6]
*Tylodelphys clavata*	PFL1	M	*Perca fluviatilis*	Germany: River Lippe	JX986909	-	Georgieva *et al*. [1]
*Tylodelphys clavata*	CL91	M	*Coregonus lavaretus*	Germany: Lake Constance	-	JQ665459	Behrmann-Godel [13]

^a^C, cercaria, M, metacercaria; A, adult; ^b^raised in experimental infection.

Distance-based [neighbour-joining (NJ)] and model-based [maximum likelihood (ML) and Bayesian inference (BI)] algorithms were used for tree reconstruction. Prior to analyses the best-fit nucleotide substitution models were selected in jModelTest 2.1.1 [21,22] using the Akaike Information Criterion (AIC). These were the Hasegawa-Kishino-Yano model including estimates of invariant sites and among-site rate heterogeneity (HKY + I + G) for the *cox*1 dataset and the Hasegawa-Kishino-Yano model including estimates of among-site rate heterogeneity (HKY + G) for the ITS dataset. ML analyses were performed in PhyML 3.0 [23] with a non-parametric bootstrap validation based on 1,000 replicates. BI analyses were carried out in MrBayes 3.2 [24] using Markov Chain Monte Carlo (MCMC) searches on two simultaneous runs of four chains during 10^7^ generations, sampling trees every 10^3^ generations. The first 25% of the sampled trees were discarded as “burn-in” for each data set and the consensus tree topology and the nodal support were estimated from the remaining samples as posterior probability values [25]. Distance matrices (p-distance model, i.e. the percentage of pairwise character differences with pairwise deletion of gaps) were also calculated and explored with MEGA v5.

## Results

### *Diplostomum* spp. infections in fish and birds

Of the 230 fish of 19 species studied, only 15 were infected with *Diplostomum* spp.: one *Cyprinus carpio* (Cyprinidae), one *Silurus glanis* (Siluridae), three *Misgurnus anguillicaudatus* (Cobitidae) and ten *Pseudochondrostoma willkommii* (Cyprinidae). All infected fishes were collected in the Ebro Delta (Tarragona, Spain) with the exception of *P. willkomii* originating from the aquaculture centre of Villafranco del Guadiana (Badajoz, Spain) (Table 1). It is worth noting that infections with metacercariae of *Diplostomum* spp. were detected in some (*C. carpio* and *M. anguillicaudatus*) and not in other relatively well-sampled species (*Pseudorasbora parva*, *Gambusia holbrooki* and *Lepomis gibbosus*) in the Ebro Delta but also in one of the three *S. glanis* sampled in this locality. All infections with *Diplostomum* spp. in the fish from Ebro Delta were of low intensity (1 to 4 metacercariae).

All *P. willkommii* (n = 10) examined from the aquaculture centre in Villafranco de Guadiana were infected with 95–139 metacercariae. Due to the high parasite load, infections were detectable by visual examination especially in older mature fish (Figure 1B,C). The overall prevalence of infection is estimated as 60–65% with a trend of increase with fish age: 0–25% in fish during the first year; 25–50% during the second year; 50–75% during the third year; up to 90% during the fourth year.

A total of 31 fish-eating birds belonging to six species was examined (Table 2). Of these, only six gulls were infected with *Diplostomum* spp.: two *Larus argentatus michahellis* and three *L. ridibundus* originating from Ebro Delta (Tarragona) and one *L. ridibundus* from Cunit (Tarragona). Representative adult specimens of the two *Diplostomum* spp. identified in the material from gulls based on morphology, i.e. *D. spathaceum* and *D. pseudospathaceum*, and all metacercariae recovered from fish were selected for sequencing.

### Molecular identification

**Figure 2:**
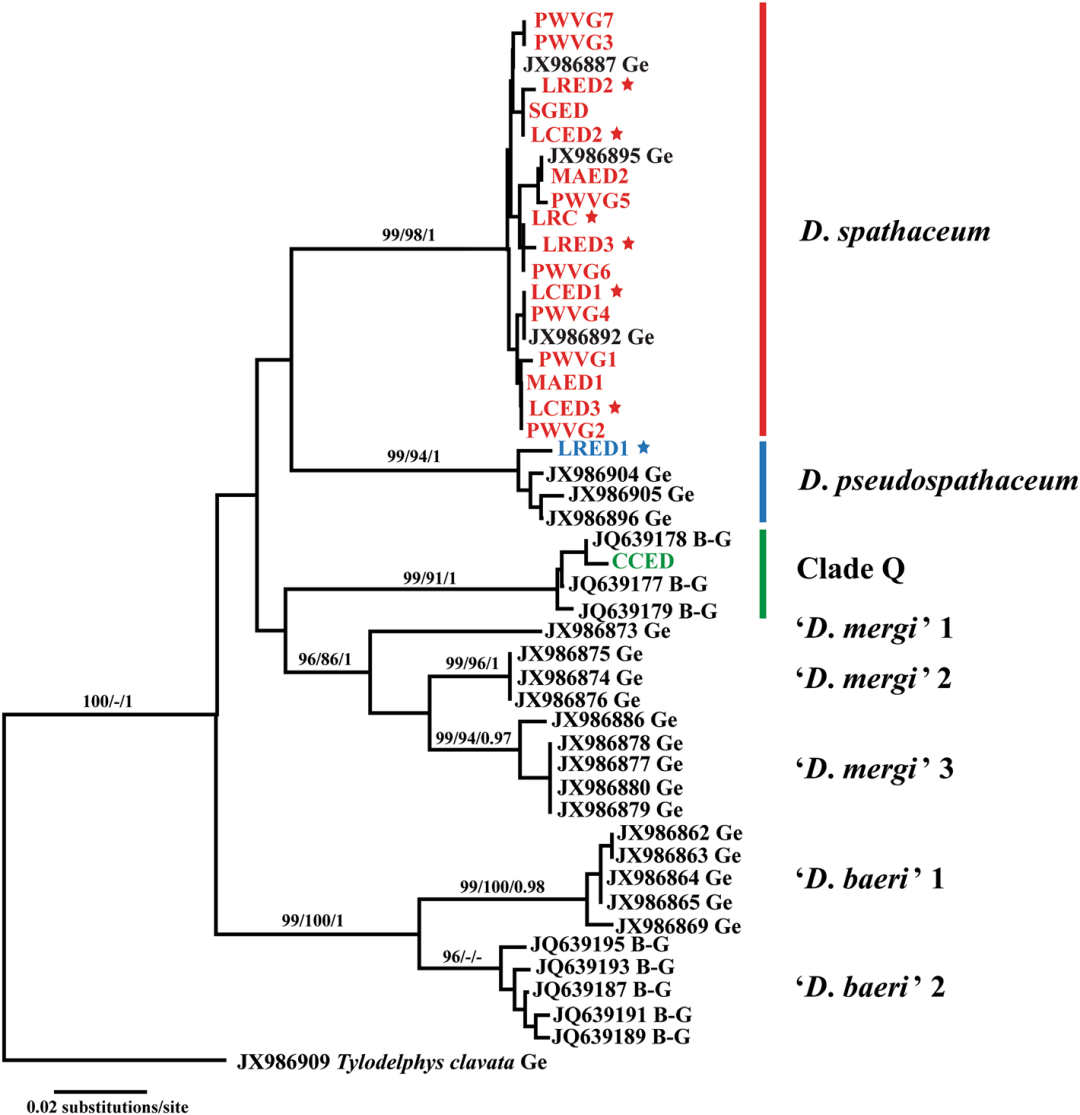
**Neighbour-joining (NJ) phylogram reconstructed using the newly-generated and retrieved from GenBank** ***cox*** **1 sequences for** ***Diplostomum*** **spp.** Nodal support from Maximum Likelihood (ML) and Bayesian Inference (BI) analyses indicated as NJ/ML/BI. Outgroup: *Tylodelphys clavata*. The scale-bar indicates the expected number of substitutions per site. Isolates from Spain are coded as in Table 3; stars indicate adult isolates from gulls.

Partial *cox*1 sequences were obtained for seven adult isolates collected from two gull hosts (*Larus ridibundus* and *L. cachinnans*) and 11 metacercarial isolates collected from the lenses of four fish hosts (*Cyprinus carpio*, *Misgurnus anguillicaudatus*, *Pseudochondrostoma willkommii* and *Silurus glanis*). Similar to a previous study on *Diplostomum* spp. in Europe [1], phylogenetic analyses of the *cox*1 dataset (410 nt) recovered eight species/lineages comprising *D. spathaceum, D. pseudospathaceum*, *D. spathaceum*/*parviventosum* referred to as Clade Q *sensu* Georgieva *et al*. [1], ‘*D. mergi*’ complex (including three putative species) and ‘*D. baeri*’ complex (representing two sibling species) (Figure 2). The analyses provided robust evidence that most of the isolates are conspecific with *D. spathaceum sensu* Georgieva *et al*. [1] (Figure 2). These represented five adult isolates ex *L. ridibundus* and *L. argentatus michahellis* from Ebro Delta, one adult isolate ex *L. ridibundus* from Cunit, seven metacercarial isolates ex *P. willkommii* from Villafranco del Guadiana, two metacercarial isolates ex *M. anguillicaudatus* and a single isolate ex *S. glanis*, the last two fish species both collected from Ebro Delta.

The intraspecific divergence within the *D. spathaceum* clade ranged between 0 and 1.5%, i.e. within the known range of intraspecific variation for *Diplostomum* spp. [1]. The material collected in Spain was represented by a total of 10 haplotypes (Table 3) with only one haplotype shared with adult isolates from central and northern Europe (haplotype 2, isolate ex *M. anguillicaudatus* and JX986892). There was no specific geographic pattern of the distribution of the novel haplotypes. Thus isolates from *Pseudochondrostoma willkommii* from the population of Villafranco del Guadiana were represented by six haplotypes with only one shared and there were shared haplotypes among isolates from geographically distant host samples, e.g. among larval isolates from Villafranco del Guadiana and adult isolates from Ebro Delta and Cunit (haplotypes 1, 3 and 4) (see Table 3 for details).

Numerous attempts were made to obtain sequences for isolates of adult *D. pseudospathaceum* identified based on morphology but only one was successful; this may be due to the fact that the infected birds were collected long after their death. The sequence for the single isolate ex *Larus ridibundus* clustered within the strongly supported clade (Figure 2) representing sequences for adult isolates of *D. pseudospathaceum* identified based on morphology [1]. The Spanish isolate was represented by a unique haplotype which differed by 1.2-1.7% from the remaining three haplotypes within the *D. pseudospathaceum* clade.

Finally, a sequence from a single metacercaria ex *Cyprinus carpio* from the Ebro Delta clustered together with sequences for one cercarial isolate ex *Radix auricularia* (RA97) and two metacercarial isolates ex *R. rutilus* (RR43 and RR45) from Lake Constance, all reported as *D. spathaceum* [13] but labelled as *D. mergi* in GenBank (see Clade Q in Figure 2).

**Figure 3:**
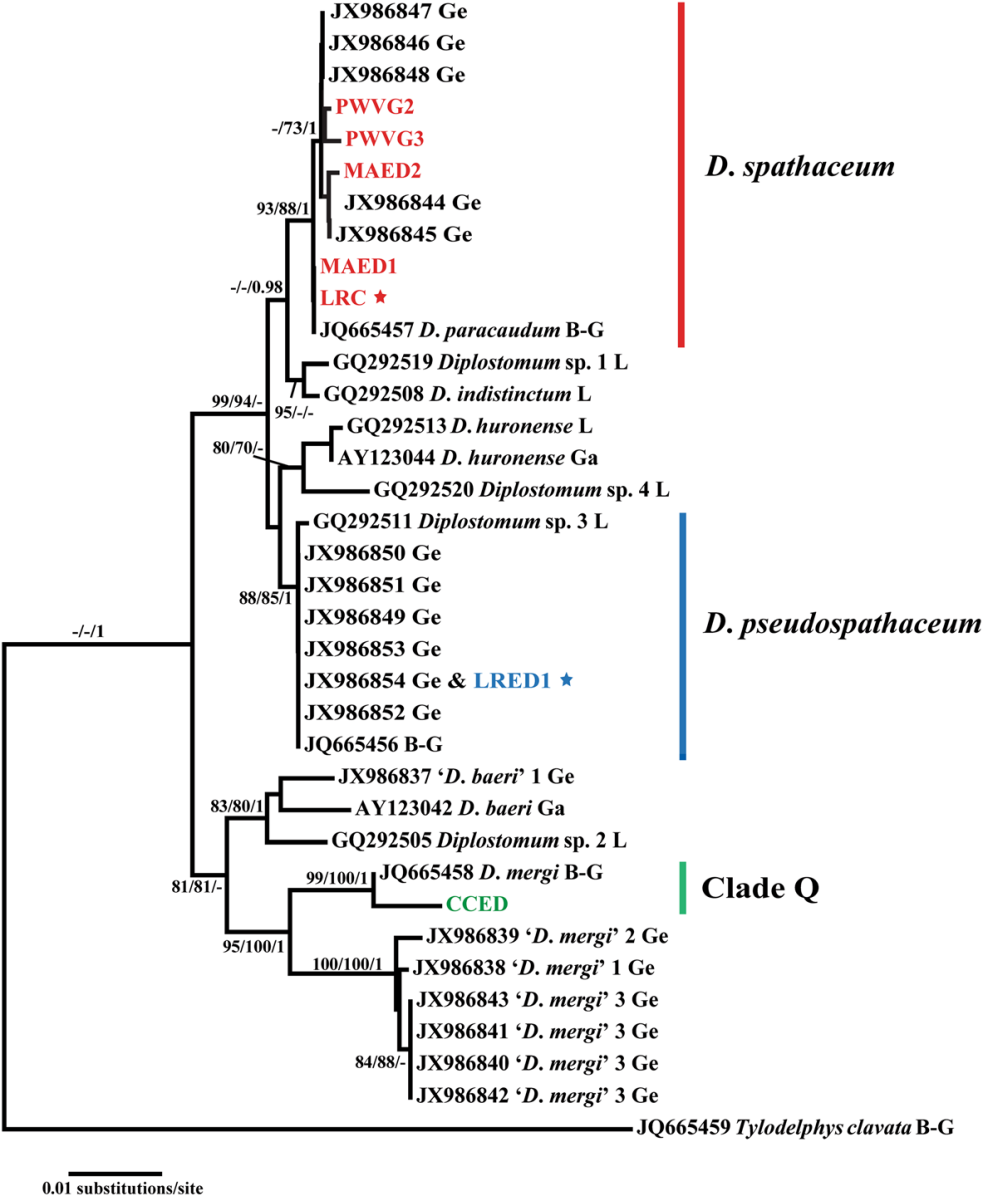
**Neighbour-joining (NJ) phylogram reconstructed using the newly-generated and retrieved from GenBank ITS1-5.8S-ITS2 sequences for** ***Diplostomum*** **spp.** Nodal support from Maximum Likelihood (ML) and Bayesian Inference (BI) analyses indicated as NJ/ML/BI. Outgroup: *Tylodelphys clavata*. The scale-bar indicates the expected number of substitutions per site. Isolates from Spain are coded as in Table 3; stars indicate adult isolates from gulls.

A total of seven ITS1-5.8S-ITS2 sequences was generated after a selective sub-sampling of the Spanish isolates within the three *cox*1 clades of *Diplostomum* spp. The analysis of the ITS data (997 nt positions) resulted in molecular identification of these isolates concordant with that based on the *cox*1 gene trees with strong support (Figure 3). The intraspecific divergence within the *D. spathaceum* clade ranged between 0 and 0.4%. The five representative isolates from the *cox*1 dataset corresponded to four genotypes (with one genotype shared between an isolate ex *M. anguillicaudatus* from Ebro Delta and one ex *L. ridibundus* from Cunit).

The sequence from the single adult isolate identified as *D. pseudospathaceum* based on morphology and *cox*1 phylogeny was identical with six sequences of Georgieva *et al*. [1] based on larval and adult isolates from the Czech Republic and Germany and one sequence of Berhrmann-Godel [13]; all these sequences formed a strongly supported clade representing *D. pseudospathaceum* (Figure 3) which also included *Diplostomum* sp. 3 of Locke *et al*. [6] as in previous studies [1,7].

As in the *cox*1 solution, the sequence for the metacercarial isolate ex *C. carpio* clustered together with a sequence labelled in GenBank as “*D. mergi*” for a cercarial isolate (RA97) ex *Radix auricularia* from Lake Constance [13] within the Clade Q *sensu* Georgieva *et al*. [1]. The divergence between the two sequences was 0.8%.

### Descriptions of the molecular voucher material

#### *Diplostomum spathaceum* (Rudolphi, 1819) (adult)

***Hosts*:***Larus argentatus michahellis* Naumann; *L. ridibundus* L.

***Localities*:** Ebro Delta, Cunit (Tarragona, Spain).

***Site in host*:** Small intestine.

**Figure 4:**
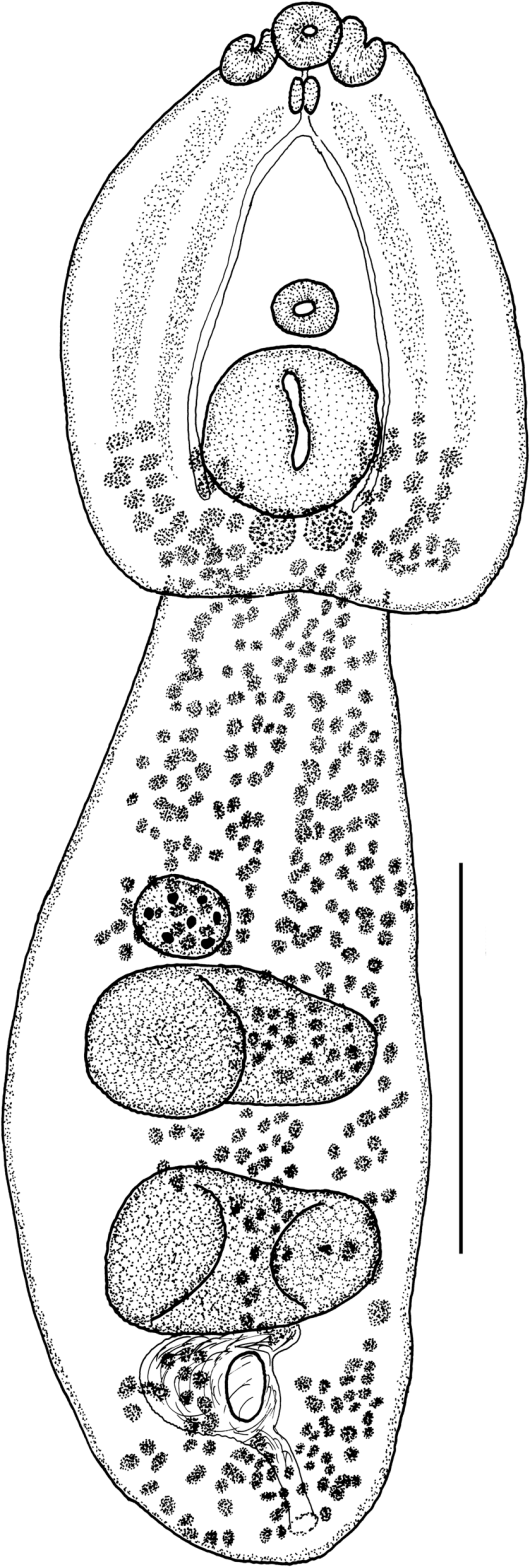
***Diplostomum spathaceum*** **.** Adult ex *Larus argentatus michahellis* (hologenophore). Scale-bar: 500 μm.

[Based on five frozen specimens (hologenophores) preserved in ethanol (molecular biology grade)]. Body 1,971 − 2,189 (2,085) long (Figure 4). Forebody oval, dorso-ventrally flattened, 782 − 1,155 long [40 − 43 (42)% of total body length], with maximum width 504 − 726 (592) at level of holdfast organ. Hindbody, elongate-oval, narrower anteriorly, 1,252 − 1,368 (1,285) long, with maximum width 387 − 575 (477) at level of anterior testis.

Oral sucker ventro-subterminal, subspherical, 71 − 93 × 70 − 92 (81 × 78). Pseudosuckers well developed, 109 − 155 × 44 − 62 (139 × 56). Ventral sucker subglobular, 65 − 95 × 80 − 99 (83 × 89), similar in size to oral sucker, located just anterior to mid-forebody. Holdfast organ large, subglobular, 150 − 236 × 202 − 288 (215 × 224), fairly close to or contiguous with ventral sucker. Prepharynx short or absent; pharynx elongate-oval, 55 − 89 × 45 − 59 (73 × 52); oesophagus indistinct; caeca narrow.

Testes 2, large, in posterior half of hindbody; anterior testis transversely elongate, asymmetrical, 171 − 203 × 154 − 224 (183 × 191); posterior testis transversely elongate, symmetrical, horseshoe-shaped, 190 − 317 × 240 − 399 (247 × 334). Seminal vesicle voluminous. Gentital pore dorso-subterminal. Ovary small, dextral, pretesticular, subglobular, 87 × 83, contiguous with anterior testis. Vitellarium follicular, follicles numerous, small, arranged in four lateral bands surrounding holdfast organ in forebody; bands reach to mid-level of holdfast organ, converge close to posterior margin of forebody, posteriorly to holdfast organ; vitelline follicles in hindbody in two wide, not well-delimited lateral bands, converging medially at level of testes, reaching fairly close to posterior extremity of body. Eggs few, 89 − 99 × 61 − 66 (95 × 63).

#### *Diplostomum spathaceum* (Rudolphi, 1819) (metacercaria)

***Hosts***: *Pseudochondrostoma willkommii* (Steindachner); *Misgurnus anguillicaudatus* (Cantor); *Silurus glanis* L.

***Localities***: Villafranco del Guadiana (*P. willkommii*) and Ebro Delta (*M. anguillicaudatus* and *S. glanis*), Spain.

***Site in host***: Eye lens.

**Figure 5:**
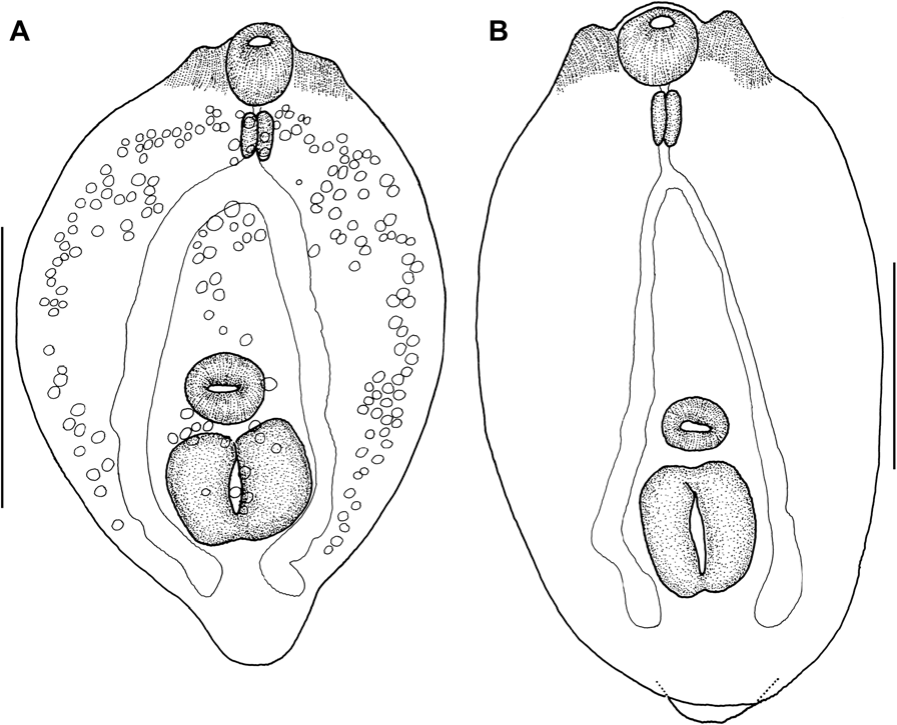
**Metacercariae of** ***Diplostomum spathaceum*** **ex** ***Pseudochondrostoma willkommii*** **.** **A**, Live metacercaria (hologenophore); **B**, Fixed metacercariae (hologenophore). Scale-bars: **A**, 200 μm; **B**, 100 μm.

[Based on 10 metacercariae (hologenophores) fixed in hot saline solution and preserved in ethanol (molecular biology grade)]. Body elongate-oval, flattened, 277 − 453 × 198 − 295 (376 × 248); primordial hindbody 10 − 26 (16) long (Figure 5). Oral sucker elongate-oval, 40 − 57 × 36 − 41 (45 × 39). Ventral sucker transversely oval, 30 − 43 × 33 − 48 (38 × 43). Two contractile lappets (pseudosuckers) present on each side of oral sucker, 44 − 55 (48) long, with maximum width 22 − 30 (26). Prepharynx very short; pharynx elongate-oval, 29 − 43 × 19 − 26 (37 × 23); oesophagus short; caeca long, wide, reach posterior to holdfast organ. Holdfast organ large, elongate-oval, 63 − 89 × 59 − 90 (75 × 80). Reserve excretory system with numerous, relatively large excretory granules (170 − 184 in number), distributed in a median and two lateral fields.

#### *Diplostomum pseudospathaceum* Niewiadomska, 1984 (adult)

***Host*:***Larus ridibundus* L.

***Locality*:** Ebro Delta (Tarragona, Spain).

***Site in host*:** Small intestine.

**Figure 6:**
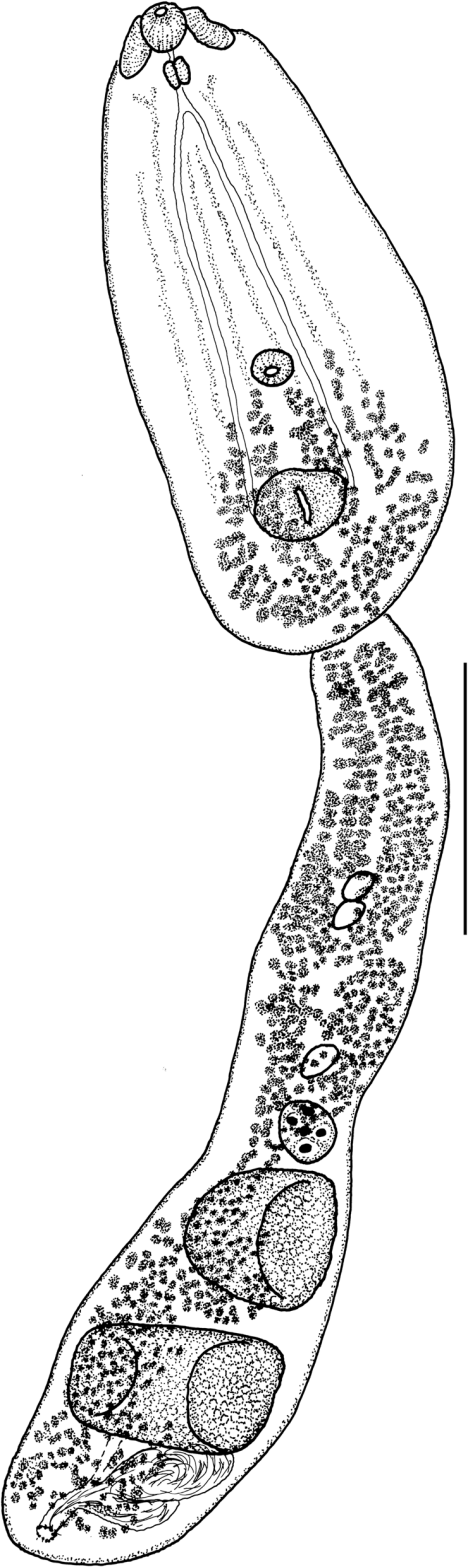
***Diplostomum pseudospathaceum*** **.** Adult ex *Larus ridibundus* (hologenophore). Scale-bar: 500 μm.

[Based on a single frozen specimen (hologenophore) preserved in ethanol (molecular biology grade)]. Body 2,884 long (Figure 6). Forebody elongate-oval, narrow, dorso-ventrally flattened, tapering anteriorly, 1,075 long (37% of total body length), with maximum width at level of ventral sucker, 526. Hindbody, elongate, sub-cylindrical, narrower anterior to ovary, 1,891 long, with maximum width at level of posterior testis, 163.

Oral sucker ventro-subterminal, subspherical, 69 × 73. Pseudosuckers well developed, 128 × 49. Ventral sucker transversely oval, 67 × 85, slightly larger than oral sucker, located just posterior to mid-forebody. Holdfast organ subglobular, 126 × 118, located well posterior to ventral sucker (at a distance >2 ventral sucker diameters). Prepharynx fairly short; pharynx elongate-oval, 53 × 35; oesophagus short; caeca narrow.

Testes 2, large, in posterior half of hindbody; anterior testis transversely elongate, asymmetrical, 132 × 75; posterior testis larger, transversely elongate, symmetrical, horseshoe-shaped, 237 × 315. Seminal vesicle voluminous. Gentital pore dorso-subterminal. Ovary small, submedian, pretesticular, subglobular, 79 × 78, nearly contiguous with anterior testis. Vitellarium follicular, follicles numerous, small, arranged in two median inter-caecal and four lateral extra-caecal bands in forebody, reaching to the posterior margin of ventral sucker anteriorly; bands, converge close to posterior margin of forebody, posteriorly to holdfast organ; vitelline follicles in hindbody in two wide, dense lateral bands, converging medially at level of gonads, reach fairly close to posterior extremity of body. Eggs few, 96 − 110 × 58 − 63.

#### *Diplostomum* sp. (metacercaria)

***Host*:***Cyprinus carpio* L.

***Locality*:** Ebro Delta (Tarragona, Spain).

***Site in host*:** Eye lens.

**Figure 7:**
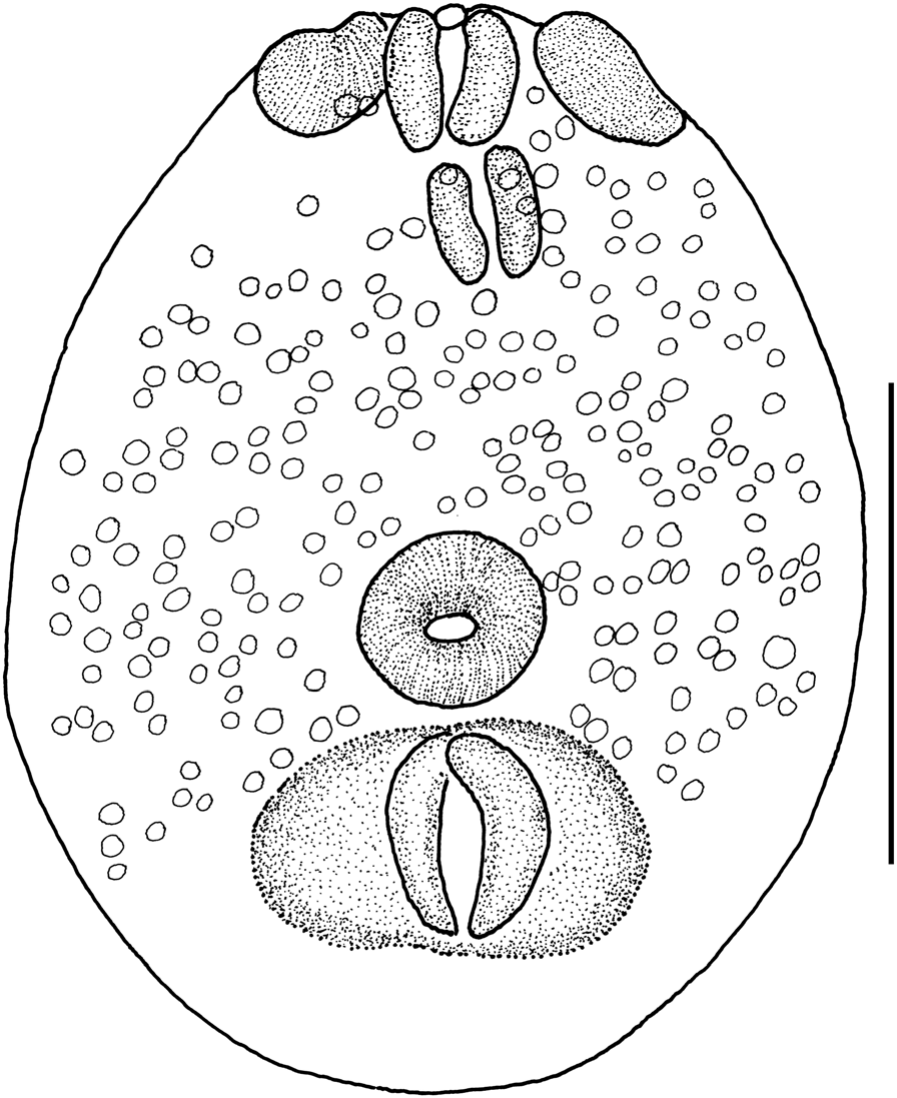
***Diplostomum*** **sp. ex** ***Cyprinus carpio*** **.** Fixed metacercaria (hologenophore). Scale-bar: 100 μm.

[Based on a single metacercaria (hologenophore) fixed and preserved in ethanol (molecular biology grade).] Body elongate-oval, flattened, 229 × 180; primordial hindbody not evident (Figure 7). Oral sucker spherical, 29 × 29. Ventral sucker subspherical, 37 × 42. Two small contractile lappets (pseudosuckers) present on each side of oral sucker, 31 − 32 long, with maximum width 15 − 16. Prepharynx absent; pharynx subspherical, 24 × 23; oesophagus very short; caeca long, narrow, reach posterior to holdfast organ. Holdfast organ large, transversely elongate, 50 × 84. Reserve excretory system with numerous, dispersed, relatively large excretory granules (c. 215 in number).

## Discussion

This first molecular exploration of the diversity of *Diplostomum* spp. in southern Europe indicates much lower species richness compared with the northern regions of Europe (3 *vs* 12 species). Of the six species of fish-eating birds studied in the north of Spain only two gull species were found to host adult *Diplostomum* spp.; however, sample sizes were rather small. The detection of metacercariae in fish also might have been influenced by the differential sample sizes. However, we found infections in an under-sampled fish host as well in some but not in other hosts with relatively large sample sizes. Notably, metacercariae of *Diplostomum* spp. were recovered in three out of the seven invasive fish species examined (*C. carpio*, *M. anguillicaudatus* and *S. glanis*; Table 1) thus indicating that these hosts may have a considerable contribution to the transmission of *Diplostomum* spp. in the Ebro Delta and elsewhere. *M. anguillicaudatus* and *S. glanis* are new host records for *D. spathaceum*.

Another important finding is the high prevalence and abundance of infection with *D. spathaceum* in *P. willkommii*, a native vulnerable species [26] with distribution restricted to the southern Iberian Peninsula in Spain and Portugal. The high levels of infections in the aquaculture centre in Villafranco de Guadiana, where mature breeders from natural populations are being added yearly to the cultured population, reveal a further threat upon this fish species in both natural and fish farming conditions. The shallow, open nature of the pools probably contributes significantly to the establishment of a focus of infection with *D. spathaceum*.

To the best of our knowledge, this study is the first to provide detailed morphometric data and morphological description of the isolates of *Diplostomum* spp. in association with the molecular data used for identification. The morphology of the adult specimens of *D. spathaceum* and *D. pseudospathaceum* used for sequence generation agrees well with the descriptions of *D. spathaceum sensu stricto* and *D. pseudospathaceum* of Niewiadomska [27], respectively. The material of *D. spathaceum* ex *Larus* spp. from Ebro Delta is characterised by lower values (outside the lower range for the material ex *Larus fuscus* L. and *L. ridibundus* from Poland studied by Niewiadomska [27] for the size of the hindbody, holdfast organ, ovary and testes (Table 5). Similarly, the specimen of *D. pseudospathaceum* ex *L. ridibundus* from Ebro Delta had smaller holdfast organ, ovary and testes and much narrower hindbody and longer pseudosuckers compared with the specimens from the same host studied in Poland (Table 5). These data indicate much higher geographic variation in the morphometric features in both *Diplostomum* spp.

**Table 5:** **Comparative metrical data for adults of** ***Diplostomum spathaceum*** **and** ***D*** **.** ***pseudospathaceum***

**Species**	***Dipostomum spathaceum***		***Dipostomum pseudospathaceum***	
**Host**	***Larus fuscus*** **L.,** ***Larus ridibundus*** **L.**	***Larus argentatus michahellis*** **Naumann;** ***Larus ridibundus*** **L.**	***Larus ridibundus*** **L.**	***Larus ridibundus*** **L.**
**Locality**	**Lake Mamry (Poland)**	**Ebro Delta (Spain)**	**Lake Mamry (Poland)**	**Ebro Delta (Spain)**
**Source**	**Niewiadomska [** 27 **]**	**Present study**	**Niewiadomska [** 27 **]**	**Present study**
TL	up to 4,000	1,971 − 2,189	up to 3,600	2,884
FBL	1,110 − 1,480	782 − 1,155	1,030 − 1,720	1,075
FBW	590 − 850	504 − 726	400 − 680	526
HBL	1,560 − 2,920	1,252 − 1,368	960 − 2,190	1,891
HBW	560 − 660	387 − 575	420 − 720	163
OSL	57 − 95	71 − 93	67 − 78	69
OSW	74 − 102	70 − 92	68 − 95	73
PSL	102 − 153	109 − 155	51 − 115	128
PSW	–	44 − 62	–	49
VSL	78 − 95	65 − 95	68 − 103	67
VSW	89 − 102	80 − 99	62 − 119	85
HOL	238 − 374	150 − 236	153 − 335	126
HOW	259 − 399	202 − 288	163 − 388	118
PHL	59 − 74	55 − 89	44 − 74	53
PHW	51 − 74	45 − 59	47 − 66	35
ATL	185 − 540	171 − 203	188 − 503	132
ATW	421 − 629	154 − 224	296 − 629	75
PTL	348 − 592	190 − 317	255 − 666	237
PTW	466 − 658	240 − 399	370 − 666	315
OVL	138 − 222	87	111 − 187	79
OVW	163 − 236	83	142 − 238	78
FO/BL (%)	31 − 48	40 − 43	41 − 58	37
Egg-length	–	89 − 99	–	96 − 110
Egg-width	–	61 − 66	–	58 − 63

*Abbreviations*: *TL* total body length, *FBL* forebody length, *FBW* forebody width, *HBL* hindbody length, *HBW* hindbody width, *OSL* oral sucker length, *OSW* oral sucker width, *PSL* pseudosucker length, *PSW* pseudosucker width, *VSL* ventral sucker length, *VSW* ventral sucker width, *HOL* holdfast organ length, *HOW* holdfast organ width, *PHL* pharynx length, *PHW* pharynx width, *ATL* anterior testis length, *ATW* anterior testis width, *PTL* posterior testis length, *PTW* posterior testis width, *OVL* ovary length, *OVW* ovary width, *FO/BL (%)* forebody as a percentage of body length.

The dimensions of the metacercariae from the three fish hosts identified molecularly as *D. spathaceum* varied within the range provided by Niewiadomska [28] for the metacercariae of this species raised experimentally in *C. carpio*. However, the mean values for the length of body and the size of suckers were lower in the specimens obtained in Spain (Table 6). The metacercaria of *Diplostomum* sp. that was found to be conspecific with the isolates of Clade Q *sensu* Georgieva *et al*. [1] had distinctly smaller oral sucker and shorter holdfast organ compared with both Spanish and Polish isolates of *D. spathaceum* (Table 6). Finally, the metacercariae of both *Diplostomum* spp. examined in Spain had distinctly lower number of excretory granules in the secondary excretory system than the experimentally raised metacercariae ex *C. carpio* (see [28]; Table 6).

**Table 6:** **Comparative metrical data for the metacercariae of** ***Diplostomum spathaceum*** **and** ***Diplostomum*** **sp. (Clade Q)**

**Species**	***Dipostomum spathaceum***				***Diplostomum*** **sp. (Clade Q)**
**Host**	***Cyprinus carpio*** **L.**		***Pseudochondrostoma willkommii*** **(Steindachner);** ***Misgurnus anguillicaudatus*** **(Cantor);** ***Silurus glanis*** **L.**		***Cyprinus carpio***
**Locality**	**Experimental infection**		**Villafranco del Guadiana and Ebro Delta (Spain)**		
**Source**	**Niewiadomska [** 28 **]**		**Present study**		**Present study**
	**Range**	**Mean**	**Range**	**Mean**	**n =1**
BL	340 − 451	398	277 − 453	376	229
BW	170 − 296	217	198 − 295	248	180
HL	–	–	10 − 26	16	0
OSL	42 − 54	48	40 − 57	45	29
OSW	42 − 52	45	36 − 41	39	29
PSL	–	–	44 − 55	48	31 − 32
PSW	–	–	22 − 30	26	15 − 16
VSL	39 − 56	46	30 − 43	38	37
VSW	42 − 59	53	33 − 48	43	42
PHL	25 − 39	31	29 − 43	37	24
PHW	12 − 25	20	19 − 26	23	23
HOL	68 − 93	77	63 − 89	75	50
HOW	62 − 102	85	59 − 90	80	84
No. of excretory granules	c. 300	–	170 − 184	178	c. 215

*Abbreviations*: *BL* body length, *BW* body width, *HL* primordial hindbody length, *OSL* oral sucker length, *OSW* oral sucker width, *PSL* pseudosucker length, *PSW* pseudosucker width, *VSL* ventral sucker length, *VSW* ventral sucker width, *HOL* holdfast organ length, *HOW* holdfast organ width, *PHL* pharynx length, *PHW* pharynx width.

Although the molecular and morphological identification of the larval and adult isolates of *D. spathaceum* and *D. pseudospathaceum* were straightforward, we failed to identify one isolate recovered in *C. carpio*. The analysis of both *cox*1 and ITS1-5.8S-ITS2 sequences placed this isolate within the Clade Q (i.e. questionable), a label used by Georgieva *et al*. [1] to indicate five identical ITS1 sequences from Europe: two for cercariae ex *R. ovata* identified as *D. spathaceum* and one for cercariae ex *R. ovata* identified as *D. parviventosum* by Niewiadomska & Laskowski [29] in Poland; one for a metacercaria ex *R. rutilus* from Finland submitted to GenBank as *D*. cf. *parviventosum*/*spathaceum* by Rellstab *et al*. [30]; and one for cercariae ex *R. auricularia* (isolate RA97) from Lake Constance [13]; the latter was designated as *D. spathaceum* but submitted to GenBank as *D. mergi*. Using the sequences of Behrmann-Godel [13] for both *cox*1 and ITS1-5.8S-ITS2, we found that this clade, incorporating our sequence for the metacercaria ex *C. carpio*, is strongly supported and reconstructed as sister to the species-level lineages of the ‘*Diplostomum mergi*’ species complex *sensu* Georgieva *et al*. [1]. Unfortunately, no identification to the species level can be attempted for the isolates within this clade since all represent larval stages for which, with the exception of the present data, no morphological evidence has been provided. The congruent morphological and molecular identification of the adult isolates of *D. spathaceum* achieved here, supports the suggestion of Georgieva *et al*. [1] that isolates in Clade Q may represent *D. parviventosum*. Further molecular and morphological evidence is required, preferably based on adult isolates, in order to solve the species-level identification of this clade.

## Conclusion

This first molecular exploration of the diversity of *Diplostomum* spp. in southern Europe indicates much lower species richness compared with the northern regions of Europe.
